# Treatment of Cholera with Iodine

**Published:** 1885-02

**Authors:** 


*Treatment of Cholera with Iodine.*—According to Dr. Senise, of Naples, iodine has an anti-choleric action *par excellence.* He asserts that cholera has never reappeared where he used tinct. of iodine, sulphuric acid and sulphurous acid as disinfectants.

As a prophylactic he ordered a teaspoonful of cognac with a drop of tr. of iodine and two to four drops of laudanum two to three times a day. Whoever used this mixture was never attacked with cholera. Professor De Renzi gives one drop of the tincture to stop the intense vomiting. He reports many cures with the iodine treatment. Maurice in a recent communication states that he has observed on the field of the microscope that the microbes while resisting to solution of 10 per cent. of carbolic acid, perish instantaneously under iodine, although used a 1 per cent. solution.

